# Vertigo-Associated Vomiting: Acute Presentation of Thiamine Deficiency in Intestinal Failure

**DOI:** 10.1097/PG9.0000000000000226

**Published:** 2022-07-25

**Authors:** Xiaoyi Zhang, Vincent Arnone, Kimberly Ackerman, Feras Al-issa

**Affiliations:** From the * Division of Pediatric Gastroenterology, Hepatology, and Nutrition, University of Pittsburgh Medical Center Children’s Hospital of Pittsburgh, Pittsburgh, PA; † West Virginia University, School of Medicine, Department of Neurology, Morgantown, WV.

**Keywords:** parenteral nutrition, Wernicke encephalopathy

## Abstract

Thiamine deficiency can manifest as Wernicke encephalopathy, with the classic clinical triad of altered mental status, nystagmus, and ataxia. Although a rare diagnosis in pediatric patients within developed countries, gastrointestinal disorders that impair nutritional intake and absorption can place patients at higher risk. Rapid diagnosis and early empiric treatment of Wernicke encephalopathy is paramount due to high risk of mortality and long-term morbidity. We present a patient with intestinal failure who developed thiamine deficiency following weaning off parenteral nutrition with acute onset of vertigo-associated vomiting. In the absence of consensus guidelines for treatment dosing and duration, in both adult and pediatric populations, we review prior pediatric cases and propose a strategy for dosing with symptom-guided step-up approach to maximize treatment efficacy in a time-conscious manner.

**What Is Known**

- Wernicke encephalopathy (WE) is a serious neurologic disorder resulting from thiamine deficiency and can occur with impaired nutritional intake or gastrointestinal absorption.
- In patients dependent on total or partial parenteral nutrition, daily supplementation of thiamine, ascorbic acid, pyridoxine, and folic acid should be maintained if parenteral multivitamin preparations are unavailable, with preference for parenteral formulation due to risk of malabsorption.

**What Is New**

- Patients with intestinal failure utilizing enteral supplements are at increased risk of micronutrient deficiencies as they wean parenteral nutrition, due to variable multivitamin preparations.
- In the absence of consensus guidelines, we suggest initial intravenous dosing of 50–100 mg, increasing toward maximum adult dosing, for example, 500 mg twice daily, until symptomatic response plateaus.

## INTRODUCTION

Patients with intestinal failure require parenteral nutrition (PN) to meet fluid and nutritional requirements. Enteral autonomy achievement remains the ultimate goal. Despite enteral nutrition (EN) advancement, patients with intestinal failure carry increased nutritional deficiency risk due to impaired enteral intake, absorption, and dysmotility. In this case, we describe the acute presentation of Wernicke encephalopathy (WE) following EN with incomplete multivitamin supplementation, review prior pediatric cases, and propose a symptom-guided dosing strategy.

## CASE PRESENTATION

A 2-year-old female with short bowel syndrome secondary to gastroschisis was admitted for postprandial emesis and decreased stool output without respiratory or other viral symptoms. She has 15 cm of small bowel anastomosed to transverse colon and history of oral aversion, recently transitioned to regular diet supplemented with powdered calorie additive, overnight dextrose-containing intravenous fluids and lipids, and daily gummy multivitamin. She had weaned PN to 10% dextrose solution and 1 g/kg/d of lipid infused over 10 hours approximately 2 months prior. Her CDC growth z scores at presentation were weight –2.13, height –3.12, and BMI 0.61.

On initial presentation, she was afebrile with mild tachycardia, but otherwise normal physical examination. Laboratory evaluation was notable for bicarbonate 17 mM (reference values 21–31), procalcitonin 0.13 ng/mL (<0.1), with hyperbilirubinemia (total bilirubin 1.4 mg/dL [0–0.7], direct bilirubin 0.3 mg/dL [0–0.2]), and mildly elevated alanine aminotransferase (62 IU/L [0–31]), with baseline mild macrocytic anemia (hemoglobin 11 g/dL [11.5–13.5], mean corpuscular volume 87.5 fL [75–87], red cell distribution width 16.9% [11.8–15.2], and mild hypoalbuminemia 3 g/dL [3.7–4.7]). Serum lipase level was normal. Respiratory viral panel including SARS-CoV2 was negative. Central venous catheter and peripheral blood cultures were negative. Abdominal film revealed nonspecific dilated bowel without frank obstruction. She received 40 mL/kg normal saline boluses and was admitted with continued antiemetic therapy.

The following day, acidosis had improved with fluid resuscitation, but emesis persisted. She developed symmetric, vertical nystagmus, with positional emesis. Family reported 1 day of unsteady gait, with patient refusing to walk or stand independently. Given findings of vertigo and truncal ataxia, neurology was consulted.

**FIGURE 1.**
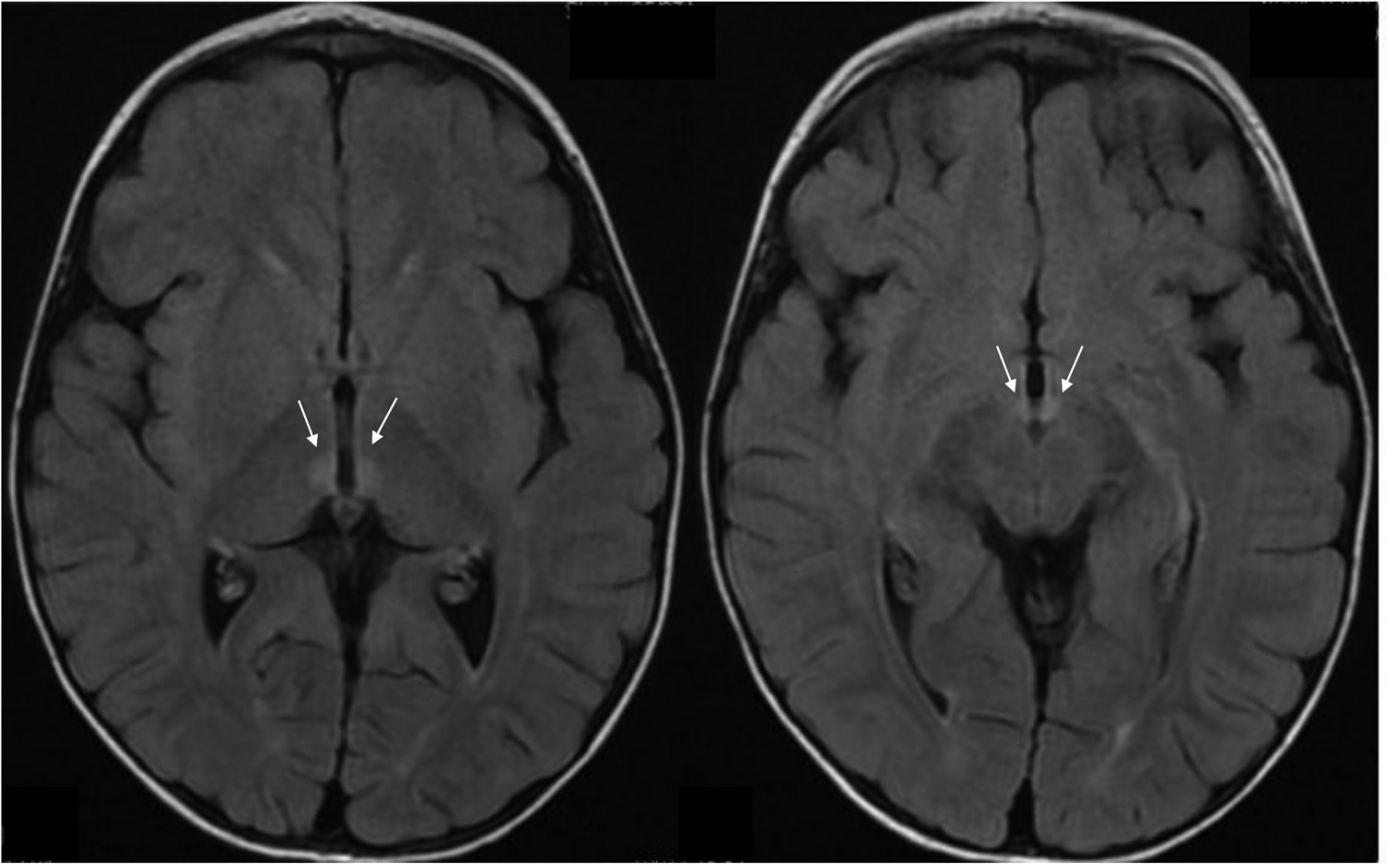
FLAIR sequence T2-weighted MRI of the brain with arrows indicating abnormal hyperintensity in the medial thalami (left) and mammillary bodies (right).

Immediate imaging included noncontrast brain MRI, whereas other causes of vomiting with associated neurologic symptoms such as meningitis, encephalitis, Lyme disease, WE, acute cerebellar ataxia, and vestibular neuronitis were evaluated with diagnostic studies including serum C-reactive protein; erythrocyte sedimentation rate; Lyme antibody titers; cerebrospinal fluid analysis including cytology, glucose, protein, HSV, EBV, and enteroviral PCRs; and serum thiamine level. Initial MRI brain without contrast was limited by motion artifact but noted patchy areas of T2 hyperintensity cortical and subcortical white matter, without evidence of intracranial mass. Repeat imaging with contrast under sedation revealed hyperintensity of the mammillary bodies and medial thalami (Figure 1), consistent with WE.

Although awaiting initial results, empiric thiamine supplementation was initiated at 50 mg intravenously daily. Further history revealed, since transitioning PN without multivitamin, she had been taking a gummy multivitamin lacking thiamine. Following MRI results with corresponding neurologic symptoms, thiamine supplementation was increased to 100 mg intravenously twice daily for three days with gradual improvement in nystagmus, and gait slowly improving thereafter. Due to residual symptoms, thiamine supplementation was increased to 200 mg 3 times daily for an additional 3 days, resulting in more substantial nystagmus and gait improvement, then decreased to 100 mg twice daily for 3 days, and transitioned to 50 mg orally twice daily as maintenance therapy. Rehabilitation teams were consulted during her admission with plans for continued early intervention home-based therapy services.

Thiamine level during admission returned <6 nM (8–30) with repeat level of 74 mM after 2 months of therapy. At 1 month following discharge, she had normal gait with mild vertical nystagmus on upgaze as her only residual neurologic symptom. Due to poor weight gain, PN was reinstated, with future consideration of bowel-lengthening procedures and glucagon-like peptide-2 therapy to achieve successful independence from PN.

## DISCUSSION

Thiamine (vitamin B_1_) is a necessary cofactor of multiple cellular metabolic pathways. Effects of thiamine deficiency on neurologic structures such as the thalamus include reduced glucose utilization, increased local lactic acid concentrations, altered cerebral blood flow, and disrupted blood-brain barrier. WE is an acute neurological disorder, most commonly occurring following glucose administration during thiamine deficiency. The classic triad of altered mental status, nystagmus or ophthalmoplegia, and abnormal gait may be absent in up to one-fifth; many are diagnosed postmortem due to under-recognition and under-treatment (1).

Commonly associated with alcohol use disorders, WE can occur with decreased nutritional intake, as in anorexia nervosa and hyperemesis gravidarum; decreased gastrointestinal absorption, such as Crohn’s disease, chronic diarrhea, and small bowel surgeries; or decreased storage capacity in end-stage liver disease. Within the context of PN, there have been reports of iatrogenic WE. The American Society for Parenteral and Enteral Nutrition has provided guidance regarding recurrent PN multivitamin shortages (2), including switching to enteral multivitamins when enteral intake exceeds 50% of needs, with caution to exclude patients with malabsorption syndromes, and carefully assessing multivitamin composition, especially with sublingual, chewable, and gummy forms. In the setting of insufficient multivitamin supply, individual daily supplementation of thiamine, ascorbic acid, pyridoxine, and folic acid should be maintained. Even with enteral intake, patients with intestinal failure are at high risk of micronutrient deficiencies due to decreased intestinal length, dysmotility, bacterial overgrowth, and high stool losses.

Classic WE MRI findings are enhanced T2 signal, signaling local tissue edema in paraventricular regions of the thalamus, hypothalamus, and periaqueductal regions (Figure 1). However, these are seen in only ~60% of patients, with low sensitivity despite high specificity for WE, often reversing quickly after initiating treatment (3). The test of choice for thiamine deficiency, erythrocyte transketolase activity with pre- and post-thiamine load measurements, is often not readily available. In the setting of mixed clinical presentations and delayed thiamine testing, neurologic symptom reversal following thiamine treatment is often the strongest early confirmation of diagnosis. If left untreated, WE can result in death or permanent neurologic impairment, and empiric treatment should always be implemented if there is clinical suspicion.

Despite agreement on early empiric treatment, consensus on dosage and duration is lacking, with adult ranges of 200 to 500 mg 3 times daily for several days or until symptom improvement plateaus. Two to three times daily dosing strategies have been suggested based on free thiamine’s blood half-life of ~100 minutes to improve brain tissue penetrance. European Federation of Neurological Societies guidelines suggest adult dosing of 200 mg 3 times daily intravenously, but note reported need for up to 500 mg in cases of alcoholism-associated WE (4). A literature search of thiamine deficiency in patients younger than 18 years of age identified over 30 case reports and series. Therapeutic treatment strategies were reviewed with representative cases and dosing regimens presented in Table 1, ranging from 25 to 300 mg, once to twice daily, via multiple delivery routes.

**TABLE 1.** Treatment regimens for pediatric Wernicke encephalopathy

Reference	Age(s)	Etiology	Dose and duration	Delivery method
Guerrero (5)	16 mo 10 mo	Malnutrition	5 mg daily 100 mg initially 10 mg daily	Hypodermic
La Selve et al (6)	12 y	Total parental nutrition	1 g × 7 d	Intravenous
Wyatt et al (7)	6 mo	Malnutrition, incomplete formula	100 mg BID	Enteral
Kalmanchey et al (8)	15 y	Osteosarcoma	300 mg/d once	Intravenous
Ming et al (9)	11 y	Chronic vomiting	100 mg/d × 14 d	Intravenous
Onodera et al (10)	12 y	Acute mixed lineage leukemia, on parenteral nutrition	150 mg/d × 3 d 100 mg/d × 5 d	Intravenous
Luxemburger et al (11)	84 infants	Thai Karen refugee population Maternal diet of polished rice	50 mg once, maternal intervention	Intramuscular
Fattal-Valevski et al (12)	9 infants 2–12 mo	Incomplete soy-based formula	50 mg/d × 14 d	Intramuscular
Muwakkit et al (13)	16 y	Acute lymphoblastic leukemia, acute pancreatitis on parenteral nutrition without supplementation	50 mg/d × 2 d	Intravenous
Darlington et al (14)	5 y	Autologous stem cell transplant, on parenteral nutrition with enteral supplement	100 mg/d × 7 d	Intramuscular
Qureshi et al (15)	23 infants 1–4 mo	Exclusively breastfed, maternal diet of polished rice	100 mg initial dose 50 mg daily	Intravenous Enteral on discharge
Bhat et al (16)	50 infants 1–6 mo	Cultural food avoidance practices, polished rice	100 mg/d 10 mg/d on discharge	Intravenous Enteral
Roilides et al (17)	9 y	Short bowel syndrome secondary to necrotizing enterocolitis, on parenteral nutrition	25 mg/d × 17 d	Intramuscular
Zhang et al (18)	6 mo	Short bowel syndrome secondary to gastroschisis, on exclusive enteral nutrition	100 mg/d × 3 d 20 mg TID × 5 d	Intravenous Enteral

In the absence of consensus guidelines and imperative need for early treatment to avoid serious morbidity and mortality, we propose a symptom-guided treatment strategy in pediatrics, with twice daily parenteral administration and interval dose increases until treatment response plateaus. Because of urinary excretion after body storage saturation, parenteral thiamine carries a minimal side-effect risk with rare anaphylaxis reports (19,20). Once clinically stable, dose can be decreased to maintenance needs, followed by outpatient laboratory monitoring. This strategy allows for rapid maximal therapeutic benefit in absence of readily available thiamine levels.

This case highlights the risk of micronutrient deficiencies in patients with intestinal failure, particularly as EN is advanced. In addition to twice yearly monitoring of fat-soluble vitamins, thiamine, B12, copper, zinc, selenium, manganese, chromium, carnitine, and fatty acids, growth must be carefully monitored with consideration of parenteral supplementation.

## ACKNOWLEDGMENTS

Written informed consent was obtained from the parent of the patient for inclusion of the case details and preparation for publication.
